# Molecular Processes Involved in the Shared Pathways between Cardiovascular Diseases and Diabetes

**DOI:** 10.3390/biomedicines11102611

**Published:** 2023-09-23

**Authors:** Julita Tokarek, Emilian Budny, Maciej Saar, Kamila Stańczak, Ewa Wojtanowska, Ewelina Młynarska, Jacek Rysz, Beata Franczyk

**Affiliations:** 1 Department of Nephrocardiology, Medical University of Lodz, ul. Zeromskiego 113, 90-549 Lodz, Polandkamila.stanczak1@stud.umed.lodz.pl (K.S.); ewa.wojtanowska@op.pl (E.W.);; 2 Department of Nephrology, Hypertension and Family Medicine, Medical University of Lodz, ul. Zeromskiego 113, 90-549 Lodz, Poland

**Keywords:** diabetes mellitus, cardiovascular diseases, microbiota

## Abstract

Cardiovascular diseases and diabetes mellitus are currently among the diseases with the highest morbidity and mortality. The pathogenesis and development of these diseases remain strongly connected, along with inflammation playing a major role. Therefore, the treatment possibilities showing a positive impact on both of these diseases could be especially beneficial for patients. SGLT-2 inhibitors and GLP-1 receptor agonists present this dual effect. Moreover, the hostile composition of the gut microbiota could influence the progression of these conditions. In this review, the authors present the latest knowledge on and innovations in diabetes mellitus and CVD—with the focus on the molecular mechanisms and the role of the microbiota.

## 1. Introduction

Currently, both cardiovascular disorders and diabetes are among the diseases with the highest morbidity and mortality score, which are becoming an increasingly significant burden all over the world [1,2]. The term cardiovascular disease (CVD) includes, among others: coronary artery disease (CAD), stroke, heart failure, atrial fibrillation, and rheumatic and valvular heart disease [3]. Even though the knowledge about these diseases and the treatment options are constantly evolving, CVD remains the main cause of death worldwide [4].

The development of CVD remains strongly connected to the complications of diabetes. Metabolic disturbances occurring due to hyperglycaemia and insulin resistance induce proinflammatory phenotype and increase oxidative stress. These changes could lead to vascular and myocardial damage [5,6]. Consequently, proper prevention, diagnosis, and treatment of both diseases play a vital role in improving the quality of the patient’s life and extending the patient’s lifespan [7]. The connection between the pathophysiology of diabetes and CVD allows obtaining drugs that could have a beneficial effect on both of these conditions [6,8]. The current review focuses on the molecular mechanisms in the pathogenesis of diabetes and CVD and the molecular basis of the treatments used in these diseases.

## 2. What Is Diabetes Mellitus?

### 2.1. Definition and Types

Diabetes mellitus (DM) is a chronic disease characterised by a progressive decrease in the production of insulin or progressive tissue resistance to insulin [9,10]. These processes lead to higher blood glucose levels compared to the healthy population [11].

The American Diabetes Association (ADA) distinguishes four main types of diabetes mellitus: type 1 diabetes (T1D), type 2 diabetes (T2D), specific types of diabetes due to other causes, and gestational diabetes mellitus (GDM) [11]. Type 1 diabetes is triggered by a progressive destruction of the β-cells of the pancreas, usually as an effect of an autoimmunological mechanism, which results in a complete lack of insulin. Type 2 diabetes is characterised by progressive tissue insulin resistance [12]. Moreover, as an effect of the acceleration of the disease, patients develop a defect of insulin secretion. Usually T2D is part of a complex metabolic syndrome [13]. The development of medicine has allowed describing many subtypes of diabetes, which are collected by the ADA into a subgroup called “specific types of diabetes due to other causes”, which contains, for example: defects of β-cell function based on a genetic disorder (e.g., maturity-onset diabetes of the young (MODY) or neonatal diabetes), diabetes induced by drugs (glucocorticosteroids, medicines used after transplantation or in the treatment of human immunodeficiency virus), endocrinopathies, and diseases of the exocrine functions of the pancreas (e.g., cystic fibrosis (CF), hemochromatosis, pancreatitis) [14]. Furthermore, there are two main requirements to diagnose GDM: firstly, diabetes had not been diagnosed prior to gestation; secondly, diabetes has been diagnosed in the 2nd or 3red trimester of pregnancy [13].

### 2.2. Diagnosis

Guidelines specify that the diagnosis of diabetes should be based on one of four criteria:

1. Random plasma glucose ≥ 200 mg/dL (11.1 mmol/L) with symptoms of hyperglycaemia.
2. Oral glucose tolerance test (OGTT) with a 75 g glucose: 2 h plasma glucose (2-hPG) level ≥ 200 mg/dL (11.1 mmol/L).
3. Fasting plasma glucose level ≥ 126 mg/dL (7.0 mmol/L) in two separate tests.
4. Haemoglobin A1C level ≥ 6.5% (48 mmol/mol) [12].

Moreover, specific autoantibodies play a vital role in the diagnosis of T1D. The most-important ones are:

- Antibodies against glutamic acid decarboxylase 65 (GAD65);
- Islet cell antigen 2 (IA-2) antibodies;
- Zinc transporter 8 (ZnT8) antibodies [15].

The detection of two or more of these autoantibodies enables diagnosing Stage 1 of diabetes (normoglycaemic, presymptomatic) [15,16].

### 2.3. Molecular Mechanism of Diabetes Mellitus

**Figure 1:**
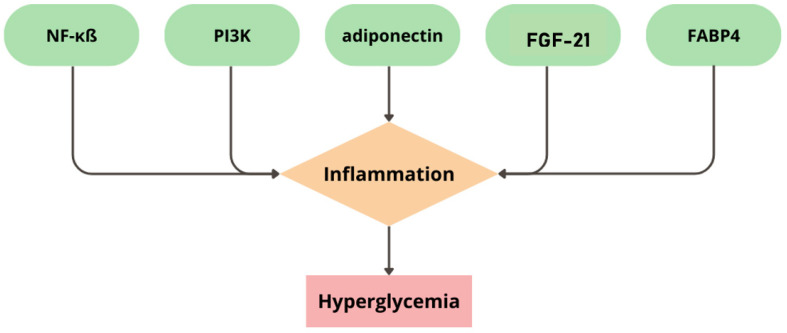
The main pathways leading to inflammation in diabetes mellitus. NF-κß—nuclear factor kappa ß; PI3Ks—phosphoinositide 3-kinases; FGF-21—fibroblast growth factor 21; FABP4—fatty-acid-binding protein 4.

Inflammation is a topic covered by many scientific studies. This pathophysiological process appears as a reaction of the human immunological system to homeostasis imbalance. Inflammation is based on the signaling pathways that could lead to the expression of pro-inflammatory cytokines and immunological response [17]. The inflammatory process could also be the basis for the course of chronic diseases, e.g., diabetes mellitus [18,19,20]. This relationship applies both to T1D and T2D, but is due to different mechanisms [21,22]. The main pathological process that causes diabetes mellitus is inflammation, consequently leading to hyperglycaemia [18,19,20]. Scientists have revealed that the main factors that cause inflammation in diabetes mellitus are disorders of the following pathways: nuclear factor kappa ß (NF-κß), phosphoinositide 3-kinases (PI3Ks), adiponectin, fibroblast growth factor 21 (FGF-21), and fatty-acid-binding protein 4 (FABP4), which are presented in Figure 1 [18,23].

Nuclear factor kappa ß causes immunological response and inflammation since this molecule upregulates the genes responsible for coding inflammatory mediators, for example: tumour necrosis factor α (TNF-α) or first and sixth interleukin (IL-1 and IL-6). Apart from inflammation, these mediators can influence the growth, survival, and development of cells [24]. TNF-α disturbs the pathway of insulin function by lowering the expression of glucose transporter type 4 (GLUT-4), which results in lower glucose absorption by cells and increased resistance to insulin [25].

Phosphoinositide 3-kinases (PI3Ks) are a group of kinases for lipids and proteins. The pathway of PI3Ks is involved in the regulation of cells’ growth and metabolism by acting through the insulin-like growth factor receptor and the insulin receptor. Moreover, by the phosphorylation of protein kinase B (Akt/PKB), this pathway could affect the survival and proliferation of cells [26]. Furthermore, by affecting glucose transporters (GLUTs), PI3Ks could be the aim of treatment resulting in higher GLUT translocation and boosting insulin sensitivity [18,27].

Adipose tissue is made of adipocytes, which secrete adiponectin. This molecule could react via receptors named AdipoR1 and AdipoR2 with substrates originally intended for insulin receptors, affecting the PI3K pathway. Moreover, adiponectin might impact liver kinase, which influences NF-κß. Therefore, these reactions could probably prevent the development of insulin resistance and, furthermore, atherosclerosis [28].

Scientific research on animal models shows that fibroblast growth factor 21 could stimulate macrophages to present an anti-inflammatory effect on adipocytes, which leads to a decreased risk of insulin resistance. Moreover, FGF-21 might increase the storage of subcutaneous adipose tissue. Consequently, this process might raise the organism’s sensitivity to insulin. Furthermore, FGF-21 could enhance adiponectin with its activity against insulin resistance [29].

Fatty-acid-binding protein 4 is a molecule responsible for transporting lipids to cells and cells’ response to them. Moreover, FABP4 could impact peroxisome-proliferator-activated receptor gamma (PPAR-γ), which regulates insulin sensitivity and adipogenesis [30]. Therefore, FABP4 might influence insulin resistance and insulin secretion in diabetes mellitus type 2 [30,31].

The vasoactive intestinal peptide (VIP) pathway plays a vital role in immunological reactions through the activation of reduced nicotinamide adenine dinucleotide phosphate (NADPH), which increases the production of reactive oxygen species (ROSs) by human phagocytes [32]. Yu et al. suggested that a lower VIP amount could be associated with the occurrence of diabetes mellitus in rodents [19].

### 2.4. Treatment

Achieving an HbA1C level under 7% is a desirable target in the treatment of diabetes mellitus [33]. Contemporary medicine gives compound therapeutic possibilities described in this section. It is worth pointing out that non-pharmacological therapy is the most-important in the treatment process of T2D. This type of diabetes is usually connected with metabolic syndrome. This leads to the conclusion that lifestyle interventions should be the main point of treatment in T2D and could be introduced with the help of. e.g., dietary and physiotherapy consultations [34]. Nevertheless, lifestyle changes alone are usually not sufficient to achieve proper glycaemic control, and pharmacotherapy is needed. The most-important groups of drugs used in the treatment of diabetes include:

1. Insulin—the main and the most-recognizable treatment of T1D and the last stage of treatment in T2D, when pancreas islets do not produce and secrete enough endogenic insulin. Adequate insulin therapy gives the opportunity to maintain the physiological level of glycaemia. The latest guidelines for treatment suggest using analogues of insulin in preference to human insulin because of their better profile of action [35,36].
2. Biguanide derivatives—with metformin as the main representative. The function of this drug is to increase the phosphorylation of the glucose transporter (GLUT), resulting in a boost of insulin sensitivity and uptake and, as consequence, leading to lover levels of glucose and HbA1c. Due to this function, metformin is used in the treatment of T2D. Moreover, metformin could support weight loss [37,38,39].
3. Thiazolidinediones (TZDs)—act through the peroxisome-proliferator-activated receptor γ (PPAR-γ), which leads to stimulation of insulin sensitivity. This group of medicines could also be used in T2D. Moreover, thiazolidinediones could decrease the patient’s weight as well [40,41,42].
4. Gliptins—work as inhibitors of dipeptidyl peptidase 4 (DPP-4), inhibiting the activation of incretin hormones such as gastric inhibitory polypeptide (GIP) and glucagon-like peptide 1 (GLP-1). These hormones are responsible for the stimulation of insulin synthesis in response to a meal. To sum up, inhibitors of DPP-4 could help to achieve better glycaemia control in patients with T2D. It is worth mentioning that these medicines have a low hypoglycaemia risk and they are safe for the cardiovascular system (CVS) [43,44,45].
5. Glucagon-like peptide 1 (GLP-1) analogues. These medicines are glucose-dependent stimulators of insulin secretion. Moreover, GLP-1 analogues lower glucagon and hepatic glucose production, which could result in better control of glycaemia. Furthermore, they could slow down digestion and decrease food intake due to lower appetite. GLP-1 analogues, similar to DPP-4 inhibitors, could be safe for CVS and stimulate weight loss [46,47,48].
6. Gliflozins—inhibit sodium–glucose co-transporter-2 (SGLT-2), resulting in lower glucose reabsorption in renal tubules and higher glucose elimination through the kidneys (by glucosuria). Scientists have described that SGLT-2 inhibitors could have a cardiorenal protective effect coexisting with normoglycaemic function. Nevertheless, these medicines could increase the risk of urinary tract infections [49,50,51].

It is also worth noting that metformin, GLP-1 receptor agonists, and SGLT-2 inhibitors have been associated with not only fat tissue reduction, but also the inhibition of epicardial adipose tissue deposition, alleviating adipose tissue inflammation, directly affecting cardiomyocytes [52].

### 2.5. Comorbidities

Diabetes mellitus, as a systemic disease, is related to abundant comorbidities.

#### 2.5.1. Hypertension

Hypertension could accompany 2/3 patients with diabetes. Moreover, it is one of the risk factors of T2D, but also, T2D increases the risk of the occurrence of hypertension. This relationship might occur as a result of pathophysiological mechanisms such as the incorrect activity of the renin–angiotensin–aldosterone (RAA) system, vascular inflammation, hyperactivity of the sympathetic nervous system, and disturbed transport of sodium in the kidneys. Furthermore, diabetes associated with hypertension increases the risk of other cardiovascular diseases [53,54].

#### 2.5.2. Obesity

Obesity is defined as abnormally high body fat deposition. The most-common obesity assessment scale is the body mass index (BMI). Obesity can be diagnosed when the BMI is over 30 kg/m^2^. Moreover, obesity is one of many modifiable risk factors of T2D. What is more, obesity could lead to insulin resistance and increase the level of inflammatory mediators, which could result in the progression of the prediabetes condition into diabetes mellitus [55,56,57].

#### 2.5.3. Polycystic Ovary Syndrome 

Polycystic ovary syndrome (PCOS) is defined as a combination of endocrinological and gynaecological disturbances, which manifests as menstrual cycle disorder and infertility problems. Scientists have proven that PCOS is a nonmodifiable risk factor of T2D. This association occurs due to the fact that patients with PCOS are at a high risk of insulin resistance, so pharmacological and nonpharmacological prevention is necessary to protect against the development of the disease [58,59].

#### 2.5.4. Dyslipidaemia

This lipid disorder is caused by abnormal metabolism and the quantity of lipoproteins such as triglycerides (TGs), low-density lipoproteins (LDLs), very-low-density lipoproteins (VLDLs), and high-density lipoproteins (HDLs). Patients with diabetes have higher amount of VLDLs and oxidative stress factors. Moreover, the simultaneous combination of diabetes and dyslipidaemia increases the risk of the occurrence of atherosclerosis [60,61,62].

#### 2.5.5. Obstructive Sleep Apnoea 

Obstructive sleep apnoea (OSA) is a disorder in which the patient presents periodic hypoxaemia during sleep as a consequence of partial obstruction of the airway. OSA is a risk factor of hypertension, cardiovascular diseases, and type 2 diabetes. The mechanism of the increased risk of diabetes is based on insulin resistance and chronic inflammation. Furthermore, recurring hypoxaemia induces higher catecholamine production and activity, which results in lower tissue sensitivity to insulin [63,64,65].

#### 2.5.6. Non-Alcoholic Fatty Liver Disease 

Non-alcoholic fatty liver disease (NAFLD) is characterised by the increased storage of fat in the liver with no other concomitant causes. A biopsy is necessary to diagnose this condition. This disease could also lead to insulin resistance, but the specific relation and mechanism remain unclear [66,67,68,69].

The comparison between risk factors and the associated molecular pathways involved in the development of DM and CVD is presented in Table 1.

### 2.6. Morbidity of Diabetes Mellitus

Diabetes mellitus is one of the most-pervasive diseases all over the world [18,85]. In 2013, the authors established that there were approximately 382-million people suffering from diabetes worldwide [86]. In 2017, 451-million people had diabetes [87,88]. Moreover, the projection of the morbidity of diabetes mellitus has been included in scientific research. A progression to 591.9-million in 2035 and 693-million in 2045 in the 18–99-year-old group of people has been forecasted [86,87]. The growing amount of people with diabetes mellitus creates a disquieting perspective regarding the progression of mortality and morbidity, which could generate higher financial burdens on the healthcare system [89].

## 3. Cardiovascular Diseases in Patients with Diabetes

**Figure 2:**
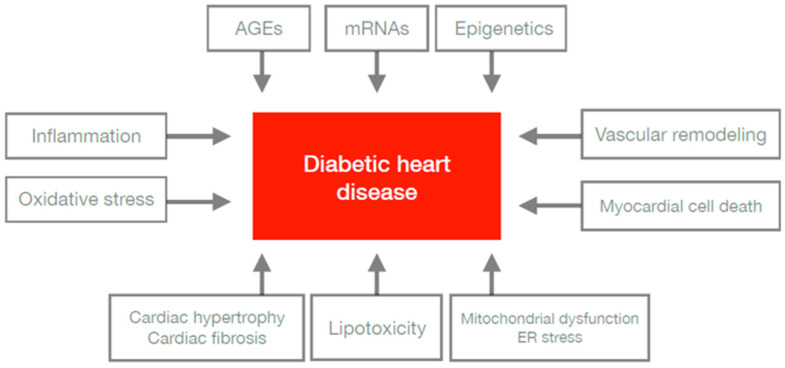
Factors affecting the development of diabetic heart disease. Factors affecting the development of diabetic heart disease: inflammation, increase of oxidative stress, increase of AGEs, expression of mRNAs, occurrence of epigenetic factors, vascular remodelling, imbalance of cell death, dysregulation of mitochondria, ER stress, lipotoxicity, cardiac hypertrophy, and cardiac fibrosis are included in the pathophysiology of cardiovascular diseases in patients with diabetes mellitus. AGEs—advanced glycation end products; mRNAs—messenger ribonucleic acids; ER—endoplasmic reticulum.

Patients living with diabetes are two-times more likely to develop and die from cardiovascular diseases (CVDs), such as myocardial infarctions, strokes, and heart failure, compared with non-diabetic subjects both in type 1 and 2 diabetes [90,91]. Heart diseases in diabetes may be characterised by an ischaemic etiology, leading to microvascular and macrovascular complications, as well as a muscle function disorder called diabetic cardiomyopathy [92,93,94]. Many studies have provided evidence for molecular changes, which, in combination, may affect the structure and function of the circulatory system. Metabolic anomalies, such as hyperglycaemia, hyperlipidaemia, inflammation, and insulin resistance, initiate a series of molecular events in the cardiovascular system involving structures such as vessels and the myocardium [5,93,95]. CVD is interlinked with diabetes due to the association with various mechanisms’ pathophysiologically. All undermentioned factors affecting the development of diabetic heart disease are presented in Figure 2.

### 3.1. Inflammation and Vascular Remodelling

Increased inflammatory cytokines, such as C-reactive protein, interleukin-6 (IL-6), interleukin-8 (IL-8), tumour necrosis factor α (TNF-α), and endothelin-1, are assumed to cause endothelial injury and coagulation dysregulations, resulting in an increased risk for CVD [70]. These molecules can exacerbate systemic insulin resistance and contribute to cardiac insulin resistance mediated by insulin receptor substrate protein-1 (IRS-1) serine (Ser) phosphorylation [71]. Increased perivascular and intermyofibrillar fibrosis has been also observed in myocardial samples in the absence of coronary artery disease and hypertension [96]. Studies on animal models of insulin deficiency revealed further increased myocardial inflammation due to increased macrophage activation [97,98]. Other studies demonstrated intramyocardial inflammation including increased expression of cell adhesion molecules: intercellular adhesion molecule-1 (ICAM-1) and vascular cell adhesion molecule 1 (VCAM-1), and an increased concentration of inflammatory cytokines and leukocytes [70,99]. Furthermore, hyperinsulinaemia promotes hepatic synthesis of prothrombotic factors and quantitative modifications in clotting factors, which combined, increase the thrombosis risk [100].

### 3.2. AGEs

It is worth mentioning advanced glycation end products (AGEs), which are advanced end products of protein glycosylation and are formed as a result of a multi-stage process that may disable protein function. Hyperglycaemia may impair the protein degradation process, collagen included, which leads to increased fibrosis and myocardial stiffness [101]. AGEs bind to the AGE receptors (RAGEs), whose expression increases in diabetic hearts due to oxidative stress [102]. AGEs also release key prosclerotic and proinflammatory cytokines and increase vascular stiffness. All these phenomena can be used to partly explain the atherosclerosis and diastolic dysfunction noted in diabetic patients [101,102].

### 3.3. Oxidative Stress

Hyperglycaemia-induced superoxide overproduction in the mitochondrial electron transport chain is thought to be a significant factor in diabetic vascular complications. The enhanced activity of nicotinamide adenine dinucleotide phosphate (NADPH) oxidase has been linked to increased reactive oxygen species (ROS) production in diabetic cardiomyocytes. Uncoupling of mitochondrial adenosine triphosphate (ATP) synthesis from oxygen intake, followed by the leakage of the mitochondrial electron transport chain, may result in decreased heart function [82]. Uncoupling of nitric oxide synthase and activation of protein kinase C, lipoxygenase, and xanthine oxidase are all sources of ROSs [103]. The creation of the potent oxidant peroxynitrite causes DNA, protein, and lipid damage, as well as the activation of stress-sensitive pathways [82].

The main metabolic pathways induced by hyperglycaemia include the polyol pathway, the hexosamine pathway, the protein kinase C (PKC) pathway, and the advanced glycation end products (AGE) pathway. These processes could not only lead to increased ROS production, but also activate inflammatory response, alter gene expression, or induce osmotic and oxidative stress [104].

### 3.4. Lipotoxicity and Mitochondrial Dysfunction ER Stress

Fatty acid (FA) absorption and oxidation overwork diabetic hearts. Increased FA oxidation has been linked to higher myocardial oxygen demand and lower cardiac efficiency [82]. This is due to FA-induced mitochondrial uncoupling of adenosine triphosphate (ATP) production from oxygen consumption, which results in energy loss. In addition, cardiac lipotoxicity caused by myocardial accumulation of lipids impairs cardiomyocyte activity. Diabetes studies on animals showed increased lipid accumulation and usage [105]. An important underlying mechanism for lipotoxic damage may involve an increase in apoptotic cell death, ceramide biosynthesis, and ROS production, as well as remodelling of the mitochondrial membrane phospholipid composition, including a decrease in cardiolipin content and an increase in the endoplasmic reticulum (ER) saturated lipid content, resulting in ER stress [106,107].

### 3.5. Myocardial Cell Death

Cardiomyocyte injury in diabetes is also promoted by altered cell homeostatic processes such as autophagy. Autophagy is a physiological process in which damaged cell components, such as organelles, proteins, and metabolites from the cell, are removed or recycled. Any dysregulations of this process lead to an imbalance in cell homeostasis [108]. Not only repression, but also an increase of autophagy have been reported in diabetic hearts and their cardiomyocytes [73,109,110]. The activation of autophagy may be insufficient in the load-stressed heart and during postischaemic reperfusion [109]. Recent findings have provided compelling evidence that insulin signaling is an important regulator of myocardial autophagy [73]. More studies are required to elucidate whether autophagy is detrimental in diverse models of diabetes.

### 3.6. Renin–Angiotensin–Aldosterone System 

Hyperglycaemia promotes angiotensinogen and angiotensin II production by activating p53 [86]. Angiotensin II directly increases vascular reactive oxygen species (ROS) production and reduces endothelial nitric oxide (NO) production. Both angiotensin II and aldosterone directly prompt oxidative stress by increasing NADPH oxidase activity [86]. Angiotensin II and aldosterone levels have also been implicated in the pro-fibrotic effects observed in patients with cardiac dysfunction induced by diabetes [87]. These abnormalities lead to the remodelling and fibrosis of diabetic hearts.

### 3.7. mRNA

Recently, micro-ribonucleic acids (miRNAs) have been defined as the micromanagers of gene expression. Many studies suggest that miRNAs take part in the pathogenesis of diabetes and cardiovascular complications such as endothelial dysfunction, angiogenesis, heart failure, and myocardial fibrosis by dysregulating the expression of multiple genes [111]. These regulators of genes are endogenous, noncoding, single-stranded ribonucleic acids (RNAs) with an average length of 22 nucleotides and are encoded by short inverted repeats within the genome [112]. Their regulation is based on the repression of translation and on promoting the degradation of target messenger ribonucleic acids (mRNAs). Changes in miRNA levels can play a crucial role in progressive dilated cardiomyopathy and heart failure [113].

### 3.8. Epigenetics

Histone modifications have also been found to play an important role in cardiac remodelling in patients with diabetes. Epigenetic factors could mediate the interplay between genes and the environment, resulting in the activation or repression of genetic transcription or even silencing genetic transcription by different types of reactions. Non-specific inhibitor-based silencing of histone deacetylases (HDACs) has been shown to promote cardiac hypertrophy and fibrosis by increasing glucose transporter-1 acetylation and mitogen-activated-protein-kinase (MAPK)-mediated phosphorylation in diabetic heart disease of mice, though their exact function should be confirmed [114,115]. Future studies may contribute to the specific molecular defects that characterise diabetic cardiomyopathy and changes in gene regulation by epigenetic mechanism and activation of transcription factors. All aforementioned factors affecting the development of diabetic heart disease are presented in Figure 2.

## 4. Drugs Used in Treatment of Diabetes and Cardiovascular Disease

The standard diabetes treatment includes different kinds of drugs, used in combination or as a monotherapy. Two of these groups, SGLT-2 inhibitors and GLP-1 receptor agonists, could be used in the treatment of both diabetes and cardiovascular disease, due to their beneficial effect on the cardiovascular system.

### 4.1. Sodium–Glucose Co-Transporter-2 Inhibitors

Sodium–glucose transporters (SGLTs) are located on the luminal membrane of the proximal tubules, where they reabsorb glucose from the glomerular filtrate. Oral SGLT-2 inhibitors (e.g., empagliflozin, dapagliflozin) are quickly absorbed into the bloodstream, where they remain for several hours. These molecules bind specifically to SGLT-2 in the luminal membrane of the early proximal tubules to reduce glucose reabsorption by 50–60% and, consequently, to reduce plasma glucose levels due to glucose excretion [116].

Although SGLT-2 inhibitors were originally developed as a glucose-lowering therapy in patients with diabetes, they present additional benefits such as lowering blood pressure [117]. Furthermore, the results of clinical trials have presented that these drugs show the ability to protect against cardiovascular disease, especially by reducing the risk of hospitalisation due to heart failure with both a reduced and preserved ejection fraction [117,118]. These beneficial effects turned out to be achievable in many subgroups of patients, regardless of diabetes diagnosis [6].

**Figure 3:**
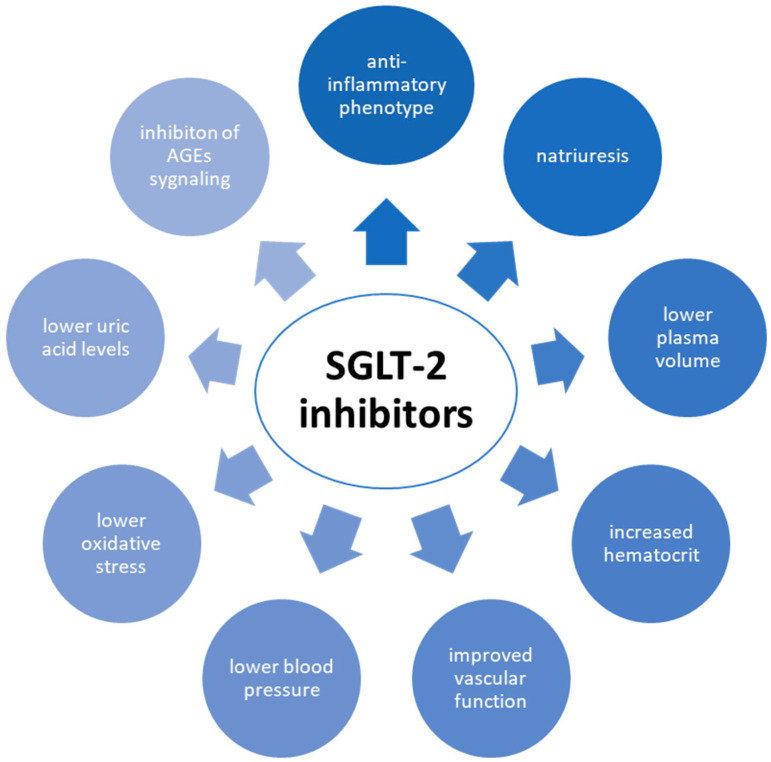
Mechanisms by which SGLT-2 inhibitors reduce the risk of CVD. SGLT-2—sodium–glucose co-transporter-2; CVD—cardiovascular disease; AGEs—advanced glycation end products.

The mechanisms by which SGLT-2 inhibitors reduce the risk of CVD are compound and multifactorial. Early natriuresis with a decreased plasma volume, a subsequent increase in haematocrit, improved vascular function, lower blood pressure, and altered tissue handling of sodium may all play a role. Other mechanisms by which SGLT2 inhibitors may be beneficial include the reduction of adipose-tissue-mediated inflammation, the lower production of pro-inflammatory cytokines, the conversion of ketone bodies into cardiac and renal metabolic substrates, the reduction of oxidative stress, the reduction of serum uric acid levels, and the inhibition of advanced glycation end product (AGE) signaling [6]. These mechanisms are presented in Figure 3.

### 4.2. Glucagon-like Peptide-1 Receptor Agonists

Glucagon-like peptide-1 (GLP-1) belongs to the group of incretin hormones, which are involved in meal-induced insulin secretion and play a vital role in maintaining normal glucose tolerance via the gut–endocrine–pancreas axis [119]. The incretin effect causes increased insulin secretion after oral glucose intake compared to parenteral glucose administration [119]. Moreover, the activation of the GLP-1 receptor might present a protective function of pancreatic islet ß cells and promote their proliferation, act as an anti-inflammatory agent, improve the function of the heart, regulate lipid metabolism, and promote nerve growth [120]. Other biological functions of GLP-1 include a reduction of appetite and delayed gastric emptying and, as an effect, might lead to weight loss [8,120].

All those metabolic changes might be beneficial to general health as well. The cardioprotective effect of GLP-1 receptor agonists combined with lowered blood pressure, improved lipid profile, and reduced body weight suggest that this group of drugs could have the potential to decrease cardiovascular risk [121]. Studies have shown that, especially, semaglutide, liraglutide, and albiglutide are able to reduce the risk of major adverse cardiac events (MACEs) [8,122,123,124].

## 5. Potential Role of Microbiome in Diabetes and Cardiovascular Diseases

In diabetes mellitus, due to the reduced blood flow combined with atheromatic malformations and sclerotic alterations in blood vessels, peripherical neuropathy occurs, which could intensify the risk of creating ulcers and wounds. A high level of blood glucose functions as nutrition for microorganisms that can infect a wound or ulcer, then overgrow and, consequently, might lead to bacteriaemia/fungaemia. In severe cases, this may cause sepsis, which could lead to septic shock. In many cases, this condition might be extremely hard to overcome, which could even lead to the demise of the patient [125]. It is also worth noticing that the human gut consists of circa 10^14^ bacteria from over 1000 species and the dysbiosis of its structure or function is observed in serious diseases such as, e.g., diabetes mellitus, Crohn’s disease, irritable bowel syndrome (IBS), obesity, and colorectal cancer [126,127].

The human gut microbiota usually consists of six main phyla of bacteria: Acinetobacteria, Proteobacteria, Firmicutes, Bacteroidetes, Verrucomicrobia, and Cyanobacteria [128,129].

Type 1 diabetes mellitus is characterised by a lower amount of mucin-degrading bacteria such as *Akkermansia municiphila*, *Bifidobacterium*, *Prevotella*, *Lactobacillus*, and *Firmicutes* and, at the same time, an increased amount of *Bacteroidetes* and *Clostridium* [130,131]. What is also important to mention is that there are reports suggesting that exposure to the Rubella virus during pregnancy can increase the chance of acquiring T1D [132].

Microbial dysbiosis in T2D consists of a *Clostridium* sp. decrease and, simultaneously, an increase of *Lactobacillus* sp., *Firmicutes* sp., and *Bacteroides* sp. [125,133]. The *Roseburia* butyrate-producing genus, as well as *Faecalibacterium prausnitzii* are decreased; however, the amount of opportunistic pathogens is increased [134].

Even though it is not clear what is the one and only cause of diabetes mellitus, microbial dysbiosis has a detrimental effect on the patient’s homeostasis in general [135].

Cardiovascular diseases (CVDs) are the leading cause of death all over the world; however, there are multiple reasons for cardiovascular diseases to occur [136]. Likewise, the alterations in the composition of the gut microbiota could exert an impact on the cardiovascular system, which might be either beneficial or detrimental [137]. Several CVDs are associated with microbial dysbiosis such as atherosclerosis, heart failure, and hypertension [137]. The influence on the human body is exerted by bacterial metabolites such as trimethylamine-N-oxide (TMAO), short-chain fatty acids (SCFAs), and bile acids (BAs), which have been shown to exert an impact on the host’s homeostasis [138].

**Figure 4:**
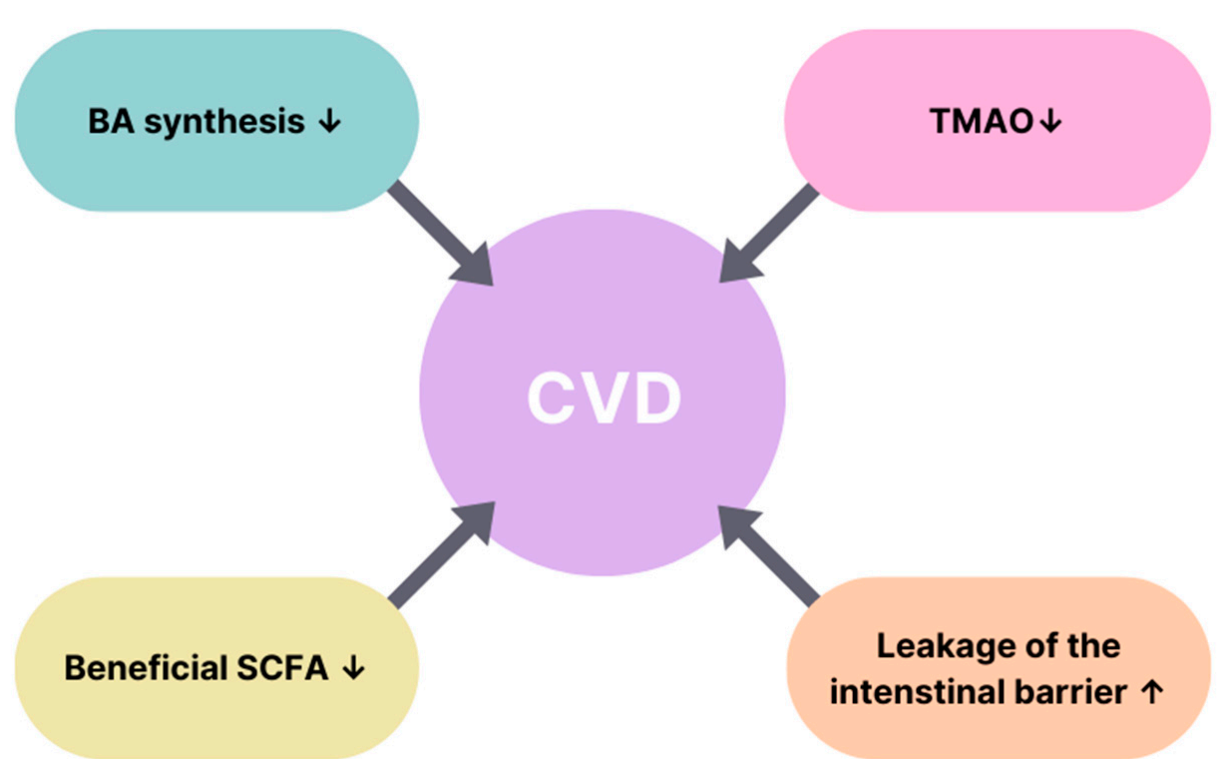
Connection between alterations of bacterial metabolites and CVDs [139]. The figure explains the correlation of factors leading to cardiovascular diseases. The increased leakage of the intestinal barrier alongside with a TMAO level decrease, the lowering of bile acid synthesis, and a reduction of beneficial SCFAs are the components that are related to the development of CVDs. ↑—increase; ↓—decrease; BA—bile acid; SCFA—short-chain fatty acid; TMAO—trimethylamine-N-oxide; CVD—cardiovascular disease.

Cardiovascular diseases can be manifested by the alteration of the microbiota’s structure and its metabolites. The decrease of faecal butyrate and the increase of the TMAO level promote the development of strokes. A decreased level of SCFA production was observed to increase the chance of both carotid artery stenosis and mesenteric ischaemia. A decrease in bile acid levels and an increase of the TMAO level were linked to promoting carotid artery disease. A reduction of bile acid synthesis and an increase of the TMAO were observed to promote peripheral artery disease [139]. Cardiovascular diseases and the associated alterations of bacterial metabolites are presented in Table 2, and the connection between the alterations of bacterial metabolites and CVDs is presented in Figure 4.

In patients with atherosclerosis, an increased number of bacteria including *Collinsella*, *E. coli*, and *Enterobacter aerogenes* has been noticed. In regard to carotid artery disease (CAD), bacteria of the genera *Lactobacillus*, *Streptococcus*, the *Escherichia/Shigella* ratio, and *Enterococcus* were increased in number, whereas the amounts of *Bacteroides*, *Faecalibacterium*, *Prevotella*, *Subdoligranulum*, *Eubacterium rectale*, and *Roseburia* were lowered. When it comes to atheromatic plaques, an increase of *Staphylococcus* sp., *Proteus vulgaris*, *Streptococcus* sp., and *Klebsiella pneumoniae* was observed. However, it is vital to mention that the change of those microbes leads to inflammation, which exacerbates atherosclerosis, but is not definitive proof of being the cause of atherosclerotic plaques’ formation. Alterations in the composition of the gut microbiota in different CVDs are presented in Table 3.

Out of many possible ways of treating diabetes and CVDs, probiotic therapy is hoped to be both effective and safe from a long-term perspective. Probiotics act by inhibition of pathogen growth, stimulation of the immune system, and changing pH levels [128]. From the great variety of microorganisms, *Sacharomycces boulardii* is one that is being used in chronic heart failure and might have the ability to lower the uric acid level, lower the level of cholesterol, and also improve the ventricular ejection fraction [143].

The most popular type of probiotics consists of the four most-common species: *Streptococcus* sp. *Bifidobacterium* sp., *Lactobacillus* sp., and *Enterococcus* sp., and their sources might be dietary such as yogurt, cheese, or fermented products, which could present beneficial effects if consumed on a regular basis [144,145].

The comorbidities leading to CVDs that could potentially be reduced by the use of probiotics are presented in Table 4 [146]. This table presents strains of probiotic bacteria that might protect against the development of diseases that lead to CVDs.

## 6. Conclusions

Diabetes mellitus (DM) is a medical condition of endocrinological origin characterised by an elevated blood glucose level, which poses a serious threat to the public health system and can be perilous to the state of wellbeing for a patient, or even lethal [133,147]. The World Health Organization (WHO) states that diabetes mellitus is also a significant stimulus for developing cardiovascular diseases such as heart failure, stroke, renal failure, gastroparesis, or Charcot joint [125]. Other comorbidities of diabetes include hypertension, obesity, PCOS, dyslipidaemia, OSA, and NAFLD.

Scientists have proven that the pathological mechanism of diabetes mellitus could mostly be based on oxidative stress and inflammatory processes. What is more, other molecular mechanisms could also participate in the progression of the disease [89]. It has been shown that many different disturbances in molecular pathways might occur and exacerbate the proinflammatory phenotype (e.g., NF-κß, PI3Ks, FGF-21, FABP4, and adiponectin).

The diabetes treatment guidelines include several groups of drugs. Nonetheless, new chances for alleviating the complications of diabetes mellitus are based on sodium–glucose co-transporter-2 (SGLT-2) inhibitors and glucagon-like peptide-1 (GLP-1) receptor agonists [127,130]. Besides, these medicines are used in the treatment of cardiovascular diseases [148,149]. This correlation is possible due to the interdependence of the pathogenesis of these diseases. Inflammation plays a significant role in the development of CVDs and DM; however, advanced glycation end products (AGEs), genetics, oxidative stress, and mitochondrial dysfunction are also involved in this compound process. Consequently, it has been found that a holistic treatment approach targeting a greater number of risk factors correlates with better CVD-free survival in patients with DM at high cardiovascular risk [150].

Moreover, the gut microbiota has a genuine effect on both diabetes and CVDs. Due to the fact that these diseases could lead to severe complications for the patient, relying entirely on probiotics may be insufficient; however, it is highly recommended to enrich the treatment with the intake of probiotics [145]. Further research on this topic is needed to fully establish the composition of the most-effective probiotic treatment in both CVDs and diabetes.

This literature review had certain limitations. Firstly, it relied entirely on previously published studies, and although great care was taken in the selection of the data included, some errors due to previously applied methods are possible. Secondly, the lack of external funding limited the availability of non-open-access research. Thirdly, only scientific papers written in English were evaluated.

A major positive outcome of this scientific study was that the key molecular mechanisms known to influence diabetes and CVDs were collected and discussed. The implementation of a treatment showing a positive impact on both diseases could be especially beneficial for patients. Therefore, further research towards the creation of novel drugs acting through common pathways of the progression of diabetes and CVDs could be the proper direction for the future.

## Figures and Tables

**Table 1:** The comparison between the risk factors and associated molecular pathways involved in the development of diabetes (DM) and cardiovascular disease (CVD).

Risk Factors	Diabetes	CVD
Inflammatory factors	increased insulin resistance by IRS-1 [70]	endothelial injury, dysregulation of coagulation [71]
Oxidative stress	increased hexosamine pathway of glucose oxidation [72]	impaired mitochondrial electron transport, damage of DNA, leading to activation of stress-sensitive pathways [73]
Epigenetics	methylation of *INS*, *PDX1*, *PPARGC1A*, and *GLP1R* [74]	hydroxymethylation of myosin heavy chain 7, inhibition of BET protein [75]
Lipid dysregulations	higher amount of VLDL [76]	increased risk of atherosclerosis [62,76]
Sleep apnoea	apnoea resulting in lower tissue sensitivity to insulin [63,64,65]	multifactorial pathophysiological processes resulting in HTN [77]
NAFLD	leading to bigger insulin resistance [68,69]	increased risk of myocardial infarction and brain stroke [78]
PCOS	higher risk of insulin resistance [76,77]	higher risk of hypertension in post-reproductive life [79]
RAAS	higher NADPH activity, resulting in cardiac dysfunction [80,81]	leading to local inflammation, which causes arterial thrombosis [82]
mRNA	m6A mRNA methylation [83]	m6A mRNA methylation [84]

NAFLD—non-alcoholic fatty liver disease; PCOS—polycystic ovary syndrome; RAAS—renin–angiotensin–aldosterone system; mRNA—messenger ribonucleic acid; IRS-1—insulin receptor substrate-1; DNA—deoxyribonucleic acid; *INS*—insulin gene; *PDX1*—pancreatic and duodenal homeobox 1 gene; *PPARGC1A*—peroxisome-proliferator-activated receptor gamma coactivator 1-alpha gene; *GLP1R*—glucagon-like peptide-1 receptor gene; BET—bromodomain and extra-terminal domain; HTN—hypertension; VLDL—very-low-density lipoprotein; NADPH—nicotinamide adenine dinucleotide phosphate.

**Table 2:** Cardiovascular diseases and associated alterations of bacterial metabolites [139].

Cardiovascular Disease	Alteration of Bacterial Metabolites
Stroke	Faecal butyrate decreased
	TMAO increased
Carotid artery stenosis	SCFA production by intestinal microbiota decreased
Mesenteric ischaemia	SCFA production by intestinal microbiota decreased
Carotid artery disease	BA decreased
	TMAO increased
Peripheral artery disease	Synthesis of BAs decreased
	TMAO increased

BAs—bile acids; SCFA—short-chain fatty acid; TMAO—trimethylamine-N-oxide.

**Table 3:** Alterations in the composition of the gut microbiota in different CVDs.

Cardiovascular Disease	Changes in the Composition of Microbiota
Atherosclerosis	*Collinsella*, *Escherichia coli*, *Enterobacter aerogenes*, *Klebsiella* sp. increased [134]
Carotid artery disease (CAD)	*Lactobacillus*, *Streptococcus*, *Escherichia/Shigella*, *Enterococcus* increased *Bacteroides*, *Prevotella*, *Faecalibacterium*, *Subdoligranulum*, *Roseburia*, *Eubacterium rectale* decreased [140,141]
Atheromatous plaques	*Proteus vulgaris*, *Staphylococcus* sp., *Klebsiella pneumoniae*, *Streptococcus* sp. increased [142]

sp.—species; CVD—cardiovascular disease.

**Table 4:** The comorbidities leading to CVDs that could potentially be reduced using probiotics.

Comorbidity	Genera of Probiotic Bacteria
Diabetes mellitus type 2	*Lactobacillus reuteri* *Lactobacillus casei* *Lactobacillus plantarum* *Bifidobacterium bifidum* *Lactobacillus rhamnosus* *Bifidobacterium lactis*
Hypertension	*Lactobacillus bulgaricus* *Lactobacillus casei* *Streptococcus thermophilus* *Lactobacillus helveticus* *Saccharomyces cerevisia*
Obesity	*Bifidobacterium animalis* sp. *lactis* *Lactobacillus rhamnosus* *Lactobacillus curvatus* *Lactobacillus plantarum*
Hypercholesterolaemia	*Lactobacillus reuteri* *Lactobacillus curvatus* *Streptococcus thermophilus* *Enterococcus faecalis* *Lactobacillus casei* *Saccharomyces boulardii* *Bifidobacterium longum* *Lactobacillus acidophilius*

sp.—species.

## Data Availability

The data used in this article were sourced from materials mentioned in the References Section.
